# Specific Roles of Lipoxygenases in Development and Responses to Stress in Plants

**DOI:** 10.3390/plants11070979

**Published:** 2022-04-04

**Authors:** Priyanka Singh, Yamshi Arif, Edyta Miszczuk, Andrzej Bajguz, Shamsul Hayat

**Affiliations:** 1 Department of Botany, Plant Physiology Section, Faculty of Life Sciences, Aligarh Muslim University, Aligarh 202002, India; singh.priyanka8156@gmail.com (P.S.); yamshiarifalig@gmail.com (Y.A.); hayat_68@yahoo.co.in (S.H.); 2 Department of Biology and Plant Ecology, Faculty of Biology, University of Bialystok, Ciolkowskiego 1J, 15-245 Bialystok, Poland; medyta93@gmail.com

**Keywords:** abiotic stress, ethylene, germination, jasmonic acid, oxylipins, phytohormone, polyunsaturated fatty acids

## Abstract

Lipoxygenases (LOXs), naturally occurring enzymes, are widely distributed in plants and animals. LOXs can be non-sulfur iron, non-heme iron, or manganese-containing dioxygenase redox enzymes. LOXs catalyze the oxidation of polyunsaturated fatty acids into fatty acid hydroperoxides. Linolenic acid, a precursor in the jasmonic acid (JA) biosynthesis, is converted to 12-oxo-phytodienoic acid through oxygenation with LOX, allene oxide synthase, and allene oxide cyclase. Moreover, JA participates in seed germination, fruit ripening, senescence, and many other physio-biochemical processes. LOXs also play crucial roles in defense responses against biotic stress, i.e., insects, pests, pathogenic attacks, and abiotic stress, such as wounding, UV-rays, extreme temperature, oxidative stress, and drought.

## 1. Introduction

Lipoxygenase (LOX) is an oxidoreductase enzyme extensively distributed in both plants and animals. The oxidation by LOX results in aroma production, and by regulating the biosynthesis of volatiles, LOX can act as a natural flavoring agent for food production. Apart from this, LOX has additional food-related appliances, including modifying dough strength and as a bleaching agent for flour [1].

In 1928, Bohn and Haas discovered LOX as an enzyme related to carotene oxidation. They observed a bleaching effect on wheat flour by adding small amounts of soy flour in the wheat dough and named the enzyme carotene oxidase, as they thought the bleaching occurred due to the oxidation of carotene [2,3]. In *Lupinus albus*, Craig [4] documented high oxygen when compared to carbon dioxide in oxygen vicinity, hence named ‘fat oxidase’ enzyme. Tauber [5] and Sumner [6] concluded that the same type of enzymes catalyzed these reactions, and after this, lipid oxidase, unsaturated lipid oxidase, and carotene oxidase were jointly named LOX. Theorell, et al. [7] were the first to extract and crystallize LOX from soybeans. LOXs are ubiquitously present in plants, e.g., they were isolated from *Arabidopsis thaliana*, *Oryza sativa*, *Zea mays*, *Vitis vinifera*, *Populus trichocarpa*, *Camellia sinensis*, *Prunus persica*, *Raphanus sativus*, *Gossypium herbaceum*, and *Capsicum annuum* [8].

This review highlights the structure and function of LOXs in plants. It also presents the mechanism of LOX-mediated reactions, the interaction of LOX with phytohormones during plant growth in abiotic and biotic stress mitigation, as well as signaling cascades in plants.

## 2. Lipoxygenases

**Figure 1:**
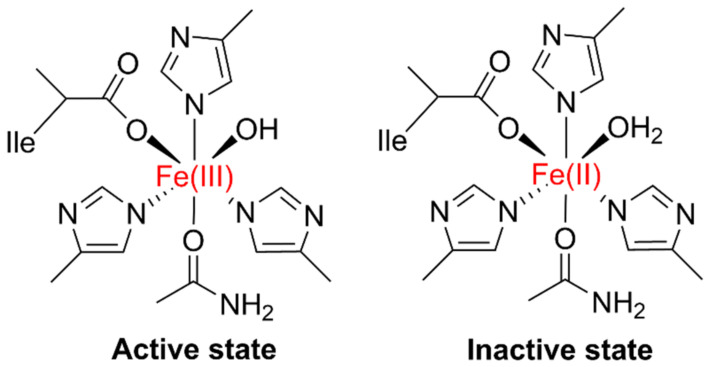
The active and inactive states of LOX. The active state possesses a spin oxidized Fe^3+^ whereas the inactive state a high-spin Fe^2+^ (reduced form).

LOXs are an assemblage of non-sulfur iron, non-heme iron, or manganese-containing dioxygenase redox enzymes with a molecular weight of 90–110 kDa; they consist of two distinct domains. The *N*-terminus of LOX is around 25–30 kDa, having a β-barrel domain, and the *C*-terminal (viz., a catalytic domain) comprises α-helix 55–65 kDa. The LOX *N*-terminal contains 160 amino acid (AA) residues, formed by two β-barrels with four antiparallel plates. The *C*-terminal has 693 AA residues, 23 α-helix, two antiparallel β-plates, and an active iron (Fe^3+^) site. For LOX, the active state is a high spin oxidized Fe^3+^ (Figure 1), and the inactive state is high-spin Fe^2+^ (reduced). In liquid oxygen’s active site, this active iron is coordinated by five AA residues, and the H_2_O molecule is the sixth ligand [9,10,11].

LOXs are structurally specific to their substrate. Esters of fatty acids and unsaturated fatty acids, with *cis* and *trans*-pentadiene structures, are suitable for LOX; in plant cells, linoleic and linolenic acids are natural LOX substrates. Various sources and LOX substrates result in oxygenation at different locations and produce their subsequent products. LOX-1 type is active with a water-soluble substrate, e.g., linoleyl sulfate, oxidation of linoleic acid gives rise to hydroperoxide at ω6 or 13-lipohydroperoxide, while the oxidation reaction of linolenic acid results in 13-hydroperoxide. While LOX-2 acts on esterification substrates to give 9 or 13-hydroperoxide, it was observed that LOX-2 and LOX-3 are more active towards triglycerides and methyl esters of fatty acids when compared with free fatty acids. A pH range of 4.5 to 8 is considered adequate for LOX activity, but it may vary in different plant species. The optimum isoelectric points for LOX-1, LOX-2, and LOX-3 are 5.68, 6.25, and 6.15, respectively [9,12,13,14].

## 3. Mechanism of LOX-Mediated Reaction

**Figure 2:**
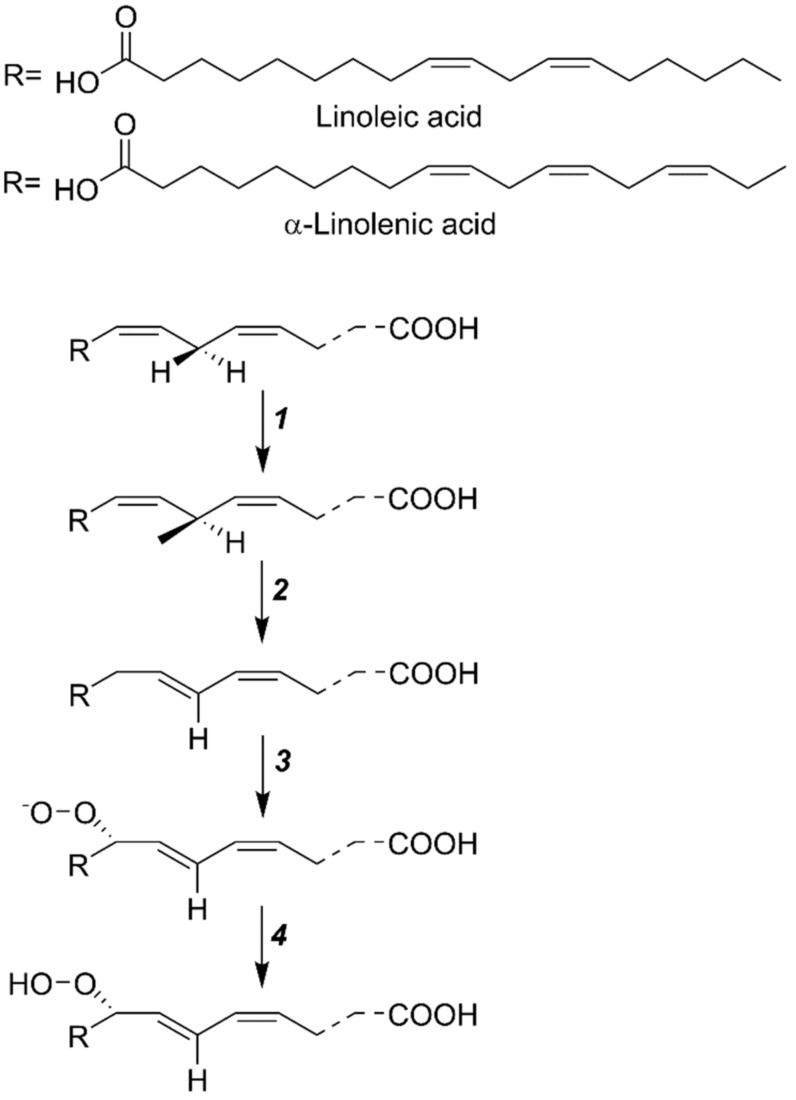
The mechanism of LOX action over the substrate: (1) removal of H^+^ ion from the substrate and the subsequent release of Fe ions, (2) reaction of molecular O_2_ with substrate-free radicals to generate peroxide free radicals, (3) transformation of O_2_ into oxygen-free radicals, and (4) reduction in peroxide-free radicals by Fe^2+^ and H_2_O_2_ formation. Finally, it becomes an active LOX enzyme.

In a wide variety of cells, the metabolic pathway of LOX initiates in plasmalemma and continues to the cytoplasm. In plants, the LOX-mediated reaction incorporates molecular oxygen at the 9 or 13-location of 18-carbon containing polyunsaturated fatty acids (PUFAs). For 9 or 13-LOX, linoleic acid (18:2) and α-linolenic acid (18:3) are the correct substrates [15]. First, linoleic acid oxygenates the LOX to produce a complex, followed by the biosynthesis of a double-free activator, viz., a hydrogen ion, and the electric ion transfer to an oxygen molecule from linoleic acid at the enzyme surface. The double-free radicals unite to make linoleic acid-hydrogen peroxide, followed by the liberation of H_2_O_2_ from the enzyme surface. Previous studies suggest that the plant-extracted LOX shows the highest activity towards linoleic acid and acts in a multistep manner (Figure 2), i.e., (1) according to the free radical theory, hydrogen atoms departure from the substrate and reduction in iron ions occur at the same time; (2) the reaction of molecular oxygen with substrate-free radicals is followed by the production of peroxide-free radicals; (3) the conversion of oxygen into the oxygen-free radicals; (4) at the climax, reduction in peroxide-free radicals by Fe^2+^ in LOX to produce H_2_O_2_, and subsequent oxidation of Fe^3+^, helps in the formation of active-LOX [12]. It is assumed that the hydrogen atoms are moved from linoleic acid to iron ions, protons, and simultaneously transferred an electron between the donor and the acceptor, hence creating an efficient hydrogen tunneling effect. It is concluded that the catalytic unit of LOX is closely related to the Fe ions. LOX shows lower activity towards methyl linoleate, linolenic acid, and arachidonic acid, while it shows no activity against oleic acid [9,13,16].

LOX can catalyze PUFAs and esters with hydroperoxide derivatives, while *cis* and *trans*-pentadiene structures, with conjugated double bonds, are synthesized by adding intra-molecular oxygen. In plants, linoleic and linolenic acid act as LOX substrates. Classification of LOXs is often categorized based on their positional specificity for the oxygenation of unsaturated fatty acids. In linoleic acid, the 9 and 13-carbon atoms in the fatty acid chain are oxygenated by 9-LOX and 13-LOX, respectively [17]. LOX catalyzes deoxygenation of region and stereo-specific (1*Z*, 4*Z*)-pentadienes into conjugated (*Z*,*E*)-diene hydroperoxy fatty acids (HPO). Degradation of HPO produces metabolites, volatile chemicals, conjugated dienoic acids, methyl jasmonate (MeJA), and jasmonic acid (JA); these composites are active participants of plant defense-related activities [18], including biotic and abiotic stress.

LOX is associated with several important biological functions, as it contributes to JA biosynthesis. In the seeds, LOX plays a crucial role in germination. LOX can act as a storage protein and can help to degrade the stored lipid bodies. The germinating seedlings of mung bean (*Vigna radiata*) possess high LOX activity; hence, these seedlings exploit as hydroperoxide lyase (HPL) enzymes. Plants are exposed to various environmental cues such as UV-rays, extreme temperature, oxygen and water shortages, insects, and pathogen attacks. These situations force the plants to induce LOX gene expression. Earlier, Leone, Melillo and Bleve-Zacheo [18] observed the role of LOX in defense of host parasites. Necrotic tissues perform more LOX activity. LOXs also participate in host-parasite interactions; however, healthy, young, and rapidly growing plant tissues show high LOX activity [19]. They are mostly situated at the outer part of the organs for serving as a barrier against pathogen attack. LOX can be expressed under the action of foreign inducers such as potassium jasmonate, chitin, and salicylic acid (SA). Generally, these inducers are either the components of the pathogen (for ex-chitin) or members of the LOX-metabolic pathway [20,21].

Lipid peroxidation is a damaging process that can harm the biofilm structure, leading to cellular dysfunction [22]. Oxidation of lipids has a dual role in organisms. In contrast, peroxyl groups’ presence interferes in the interaction of hydrophobic lipids and proteins and lipids, resulting in altered proteins and biofilm structure [23]. In the meantime, peroxyl lipids are also responsible for free radical production that further causes changes in lipid proteins and biofilms [24,25]. The oxidation of the lipid bilayer of biofilm forces the film to lose its barrier role, disturbing the subcellular structure and the entire cell. When lipid oxidation is manipulated by any mechanism in a definite cell space, it may cause a significant impact on cells and the whole organism. Lipid oxidation enhances eicosanoids synthesis that participated in lipid mobilization, cell differentiation, and cell maturation [26].

LOX-mediated fatty acid peroxide’s productions are changed to various compounds via at least six pathways under the supervision of enzymes collectively named oxylipins. Earlier investigations suggested that oxylipins are crucial biological regulators that play an essential role in plant growth, development, organ formation, senescence, signal transduction, and homeostasis. Enzymes that participate in this metabolism are mainly propylene oxide synthase, hydrogen oxidase, reductase, peroxidase, and diethylene ether synthase [27].

## 4. Regulation of LOX Signaling Associated Scheme

Modulation of phospholipase activity and desaturase enzymes, related to PUFAs production from the phospholipids, can influence LOX metabolism. JA and its derivative also affect the cytochrome oxidase (*CYP74*) and *LOX* expression [28]. Higher transcription of the allene oxide synthase (*AOS*) gene and enhanced allene oxide cyclase (AOC) activity are generally observed in wounded leaves. It was found that the promoter region of many genes belonging to the cytochrome oxidases family have elements that are responsive to the stress signals produced by hormones, such as ethylene (ET), JA, and SA. Transcription of the HPL gene is increased under pathogen attack and mechanical injuries, whereas in contrast to AOC, the enzyme biosynthesis was not promoted by MeJA application [29,30]. An instant liberation of *HPL* products just after the mechanical injury is the symbol of the plant constitute enzyme, and their biosynthesis de novo, are not mandatory under stress. Contact to the substrate after membrane destruction is responsible for *HPL* activation. In the meantime, the enzyme is located on the membrane and moved towards the injured side. The *13-HPL* genes are expressed in the above-ground plant parts, whereas *9-HPL* is expressed in the below-ground parts. The organ specificity of HPL is correlated with the suitable organ specificity of LOX, and HPL activity is correlated with the chlorophyll content. Consequently, the highest activity was reported in unripe pepper, while during ripening, the enzyme activity is considerably reduced [31]. Diverse types of cells were exposed to change in the expression of the *HPL* and *AOS* genes. The expression of the *AOS* gene occurred in tomato (*Solanum lycopersicon*) organs, while *HPL* genes were expressed merely in flowers and leaves [32]. The expression of *AOS* is tissue-specific. Regulation of JA biosynthesis is multifaceted and multistep, and the interference of elicitors, mechanical wounds, and ET can raise JA and *cis*-12-oxophytodienoic acid (OPDA) levels, thereby promoting AOS activity. ET and JA can, optimistically, influence each other’s biosynthesis during aging and in defense mechanism activation. SA stimulates AOS aggregation in dicotyledons, and in *Arabidopsis thaliana*, SA application was found to accumulate the OPDA [33,34]. JA biosynthesis restricting the SA during OPDA manufacturing is well illustrated via a rapid JA consumption in the SA-supplemented tissue, which is the pattern of localization of JA biosynthesis. The expression of the divinyl ether synthase (*DES*, a unique LOX pathway of product conversion) gene is connected with a pathogen attack [35]. The expression of the *DES* gene in the pepper (*Capsicum annuum*), infected with the *Obuda pepper* virus, was observed [36].

LOX encoding genes have been recognized in various crops, including *Arabidopsis thaliana* (6 genes), *Oryza sativa* (14 genes), *Vitis vinifera* (18 genes), *Populus trichocarpa* (21 genes), and *Capsicum annuum* (8 genes) [37]. In *Arabidopsis thaliana*, *AtLOX1* is related to the defense response of pathogen in plant leaves [38], *AtLOX2* is responsible for JA biosynthesis [39], *AtLOX3* and *AtLOX4* show a crucial role in flower development and male fertility regulation [40], *AtLOX5* participates in defense response and lateral root development [41], while *AtLOX6* is involved in JA biosynthesis and is generally expressed in roots [42] (Table 1).

The LOX proteins in *Arabidopsis thaliana* are made up of around 850–930 amino acids [43]. The information about *LOX* genes has been assembled and uploaded to the *Arabidopsis* genome database (https://www.arabidopsis.org/, accessed on 1 March 2022). Similarly, information about *LOX* genes was uploaded to (http://rice.uga.edu/, accessed on 1 March 2022). Primarily, these genes are composed of around 790–950 amino acids, but certain genes produce very small peptide chains. The four *OsLOX* genes were noted to have a chloroplast precursor (Table 2). The *OsLOX3* gene is involved in providing disease resistance in *Oryza sativa* [44].

## 5. LOX and Related Metabolites in Stress Mitigation via Phytohormonal Interaction

Several defense-related mechanisms participate in developing plant responses against both biotic and abiotic stresses. Signaling organization, triggering the production of anti-stress compounds and their activation, depends on pathogens and patterns of abiotic stress. Signaling molecules such as JA, MeJA, abscisic acid (ABA), ET, SA, and other metabolites may work either alone or interact with each other in either an antagonistic or synergistic manner [45]. They are organically caught up in a diverse signaling web, which permits plants to pertain to more appropriate defense tactics against abiotic and biotic stresses [46]. Various investigations have exposed that the action of LOX enhances mechanical injuries [47], ozone effects [48], elicitors [49], and hyperthermia [50].

**Figure 3:**
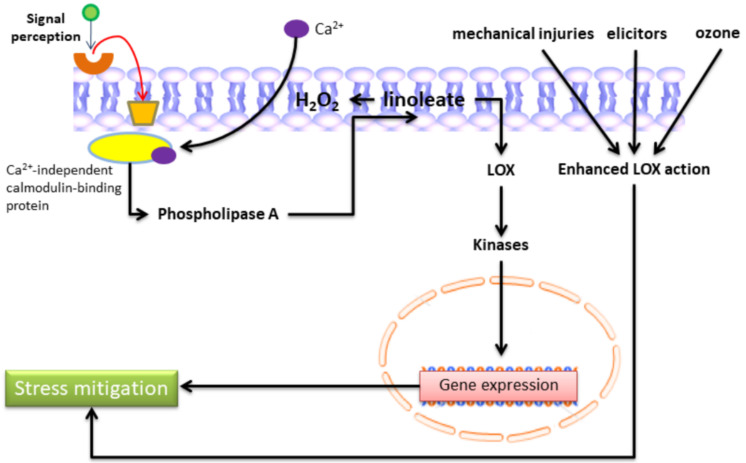
LOX in stress response signaling. The activation of phospholipase A by Ca^2+^ and calmodulin leads to the release of linoleic and linolenic acids from membrane phospholipids, which are used as substrates by LOXs. Next, hydroperoxidation of fatty acids by LOX can trigger protein kinases and induce the expression of several genes whose products are assumed to be involved in defense reactions.

LOX metabolism is further strengthened by the activation of genes encoding several important enzymes. There is sufficient data available, assuming that the LOX pathway of lipid membrane conversion is a crucial, autonomous signaling pathway [51,52]. As with other signaling pathways, primary signal interaction with the plasma membrane receptors stimulates a membrane-bound protein that signals along the signal route. Signals of the LOX pathway are increased by auto-catalytic cycles that involve Ca^2+^ and calmodulin ions. Hydroperoxide is produced in the plasma membrane from the linoleate, and linolenate transports Ca^2+^ ions from the outer side of the cell to the inner side [28]. An elevation in cytoplasmic Ca^2+^ ion content upshots the activation of phospholipase A and the liberation of PUFAs from the phospholipids [17]. Under auto-catalytic cycle conditions, MeJA expresses the genes related to desaturase, which conducts catalytic conversion of linoleic acid to linolenic acid [53]. Intermediates and end-products of LOX metabolism can trigger protein kinases, transmit signals, and produce their transduction (Figure 3). Though the molecular mechanisms related to oxylipin-mediated gene activation are not well studied, the available data suggests that the following compounds express the protein genes that participated in developing stress tolerance [45,52].

JA belongs among the well-studied oxylipins of the LOX signaling system. JA and MeJA stimulate the production of proteinase inhibitors and are participants in the release and accumulation of alkaloids, given a selective inhibition of polypeptide-synthesis [28,54]. Forming the active oxygen molecule and inducing the synthesis of few defensive compounds associated with the pathogenesis, JA stimulates resistance to plant disease [55]. It was also observed that JA is the part of signaling that came from the surface of an infected cell to the nuclear region, and in intercellular signaling, it supports the expression of protective genes [28]. A mechanically injured plant induces a JA-dependent response. Its tissues dry out. The injured portion starts accumulating ABA, expressing the *ICK1* (a cyclin-dependent protein kinase inhibitor), which further interacts with the cyclin D3 and represses the activity of cyclin-dependent kinase complexes [56,57,58]. JA and its precursors participated in the compound multi-constituent signaling pathway accountable for plant immunity. Starting segments of phytohormone signaling by external JA are supposed to be associated with regulating the transportation of Ca^2+^ and H^+^ ions via the plasmalemma [59]. It is observed that mechanical injury stimulated the JA and MeJA aggregation, and this happens along with the activation of some key enzymes implicated in the synthesis of these compounds. This aggregated JA further expresses the protective genes, including inhibitor genes of proteinases and the phenylalanine ammonia-lyase (PAL) gene [60,61]. Inactivation of genes related to the biosynthesis of JA is accompanied by a repression of the plant-protective response [62]. In tomatoes, systemin and mechanical injuries were found to express the proteinase inhibitors via a JA-included signaling pathway. JA is an outcome of the fatty acid hydroperoxide metabolic pathway using AOC [21,51]. Although it is evident that in some plants, this signaling pathway can be completed without the participation of the enzyme. It was noticed that the infected oat leaves and rice (*Oryza sativa*) shoots do not involve JA in signaling [31]. Available data revealed that molecular mechanisms of JA, under abiotic stresses, are exact and variable. This can be explained by the huge variety and number of JA-signaling elements and by having particular and overlapping roles of individual metabolites of the AOS branch. In various plants, HPL is considered a leading enzyme in the hydroperoxide metabolism of leaves, and because of this, the results of HPL activity implement the signaling roles. Enzyme *trans*-2-hexenal is reported to conduct biosynthesis of PAL, an enzyme responsible for conducting the production of a lignin predecessor and promoting cell wall thickening [63].

The map-based cloning studies disclose that tomato lipoxygenase D (*TomLoxD*), which is a 13-lipoxygenase, catalyzes the biosynthesis of JA [21,64,65,66]. According to the study of Kućko, et al. [67], oxidative stress-induced lipid peroxidation and subsequent LOX reaction resulted in JA formation in the flower abscission zone cells. Climacteric fruit ripening is a well-regulated (genetically and developmentally) process characterized by several physio-biochemical alterations, including the enhanced rate of ET production, respiration, fruit texture, aroma, flavor, and color [68]. During the ripening process, the ripening-specific *LOX* gene expression alters the level of endogenous ET [69]. Several studies have focused on the role of LOX in ET production and suggested that LOX may induce ACC oxidase under unfavorable conditions. LOX-induced free radicals transform ACC into ET [68,70]. Similarly, Yu, et al. [71] also reported the role of LOX-induced superoxide free radicals in controlling ET biosynthesis. They also observed that elevated JA levels regulated the LOX activity and subsequently influenced ET synthesis. Furthermore, high temperature, as well as JA and SA application, promote *Gllox1* and *Gllox2* expression in macroalga *Gracilariopsis lemaneiformis* (Rhodophyta). It can be concluded that JA, SA, and ET positively interact with LOX to regulate several physio-biochemical processes in plants [72].

PAL can also catalyze SA biosynthesis, helps in accumulating H_2_O_2_ (which acts as a toxin for pathogens), and produces plant antibiotics, such as phenylpropan phytoalexins. Another compound, glutathione-S-transferase, produced with the help of enzyme 4-hydroxy-2-nonenal, participates in eradicating toxic substances from the plants [73,74]. It is now well known that oxylipins make them interact with the biologically active elements of other signaling pathways, especially with phytohormones. In wounded plants, ABA was observed to positively manipulate the LOX activity [75,76]. In wounded rice (*Oryza sativa*) leaves, ABA applications were reported to trigger the LOX activity, enhance the JA level, stimulate peroxide oxidation of membrane lipids, and enhance stress tolerance [77]. A positive correlation was observed between the water-stressed plants’ ABA and LOX transcript levels [78]. Likewise, mechanical injuries increase JA, ABA, and LOX activity [79,80]. During osmotic stress, the level of LOX-1 LOX-2 mRNA is enhanced, leading to high enzymatic activity [48]. Application of 24-epibrassinolide resulted in a three to six-fold increase in the content of the product of 9-LOX. Treatment of in the cold-stressed pea (*Pisum sativum*) enhanced the LOX activity. This brassinosteroid (BR) blocks the inhibiting effect of JA on the growth of the pea root. It participates in the JA-signaling cascade, expressing the *DWF4* gene responsible for synthesizing an important enzyme involved in BR development [81,82]. SA was also reported to inhibit the synthesis of 12-OPDA, 10,11-reductase and increase the local immunity via OPDA accumulation [34,83,84].

## 6. LOX in Seed Germination and Growth of Seedling

LOXs function similarly to vegetative storage proteins (VSPs) and perform a crucial role during the maturation of seeds and seedling growth [85]. At the time of germination, different kinds of LOXs get activated for mobilizing lipids. It was observed that, during the early stage of seedling growth in several crops (e.g., *Arabidopsis thaliana*, *Vigna radiata*, *Citrullus lanatus*, rice, and *Brassica napus*), various novel LOXs are induced, whereas, in germinating soybean (*Glycine max*), degradation of lipids via a LOX-independent pathway, as the three known isoforms of LOX found in mature seeds, vanish during the early germination stage [51,83]. The proteome profiling analysis in soybean and rice germinating seedlings showed that the LOX-dependent process contributed to the degradation of lipids, and LOX can also assist in eradicating the ROS production during quick transfers of reserve materials germinating soybean seeds [84]. In soybean, a major LOX L-4 emerged in cotyledons after germination and generated (13*S*,9*Z*,10*E*)-13-hydroperoxy-9,11-octadecadienoic acid (13-HPODE) and (9*S*,10*E*,12*Z*)-9-hydroperoxy-10,12-octadecadienoic acid (9-HPODE) from the α-linoleic acid (LA). Apart from germination, rice with *OsLOX2* disturbs the seed endurance during storage [86], and suppression of this gene may delay the process of aging during storage without surrendering germination. *LOX3* gene suppression by the antisense method in rice endosperm exposed that transgenic plants exhibited an apparent reduction in the LOX transcripts levels, and low-level expression of *9-HPODE* from LA was observed [87]. In diverse bread wheat cultivars, a decline in LOX-specific activity resulted in an effective drop-off in lipid oxidation of grain and extended the grain storage time [88]. Three novel isoenzymes of LOX (P-1, P-2, and P-3) were expressed during the germination of *Vicia sativa*, and these LOX isoenzymes perform fatty acid lyase and dioxygenase activities. Lentil, kidney bean, broad bean, and chickpea seeds have two main LOXs; one mainly synthesizes 13-HPODE from the LA, and the other synthesizes 9- and 13-ketodienes and 9- and 13-HPODEs [89,90]. These LOX isoenzymes were observed to have similar biochemical activities to isoenzymes in soybeans [91]. During the seed development stage, almond and *Zea mays* showed more expression of 9-LOX [52,92]. In cucumber (*Cucumis sativus*), the physiological role of LOXs in cotyledons was checked via the shaping content of LOX-derived compounds and oxylipins [93]; this was the pioneer verification for the involvement of lipid body LOX type in volatile aldehyde production. In *Arabidopsis thaliana* seeds, resemblance in natural and aging-induced modifications in lipidome was noticed [94]. Recognition of numerous seed aging markers includes several hydroxylated triacylglycerols, diacylglycerol, phospholipids, and oxidized/non-oxidized fatty acids. It was observed that LOX1 and LOX2 are assigned for producing enzymatically and non-enzymatically oxidized free fatty acids, correspondingly. In non-seeds tissues, LOXs can be utilized for VSP and are also found in germinating cotyledons, flowers, and pod walls. VSPs show their participation in the developmental process and excess nitrogen product aggregation [95].

## 7. LOXs in Male Gametophyte

LOXs can also show their effect on the male gametophyte. In olive, during pollen grain germination, storage lipids are progressively processed when they reach the pollen tube growth stage. The functional characterization of the enzyme system of the male gametophyte during lipid mobilization exposed the phospholipase A (lipase enzyme) related to an important role of the LOX enzyme [96]. During programmed cell death of *Lathyrus undulates*, LOXs mediate the degradation of the tapetal cell membrane [97]. The transgenic experiments prove that POTLOX-1 shows the 9-LOX activity in potato roots and tubers [98]. In stored tubers, a positive correlation with LOX and tuber russet skin was observed. LOX mRNA proteins were recognized in developing legume nodules, but their action was reduced in developed nodules [27,99].

## 8. LOX in Pathogen Attack

During pathogen attack, plants start releasing metabolites with preventive and toxic effects, whereas other metabolites function as signaling molecules to stimulate defense-related responses. For biting and chewing types of herbivores, the LOX pathway plays a significant role in defense mechanisms by foremost oxylipins such as 10-OPDA (a positional isomer of 12-OPDA), 12-OPDA via the action of 13-LOXs, and 10-oxo-11-phytoenoic acid (10-OPEA) via the action of 9-LOXs [100]. Herbivory results in an anti-herbivore oxidative shift, which results in either direct or indirect oxidative damage to herbivores and transmits the signals for managing the defense mechanism of plants [101]. The 9-LOX pathway was reported to have a considerable role in providing defense against pathogen attacks, e.g., *Pseudomonas syrigae* [102,103]. Likewise, *Zea mays*, the 10-OPEA with 12 and 14-C cyclopente(a)nones (together named as ‘death acids’), have a crucial role in more JA synthesis under the infection of *Cochliobolus heterostrophus* [104]. This infection enhances phytoalexin activity, defense-related gene expression, and may boost cytotoxicity, and ultimately, cell death. The *ZmLOX4* and *ZmLOX5* are two segmentally-duplicated *9-LOX* genes of *Zea mays* and were induced under the fungal infection of *Fusarium* *verticillioides* and *Cochliobolus carbonum* to perform an exclusive resistance mechanism [102].

The 9-LOX pathway was reported to modify oxidative stresses, plant defense, and lipid peroxidation in *Arabidopsis thaliana* [105]. Even in pepper leaves, the pathogen’s attack enhances the expression of a *9-LOX* gene, *CaLOX1* [106]. High expression of *CaLOX1* in *Arabidopsis thaliana* resulted in more resistance towards *Alternaria brassicicola, Hyaloperonospora arabidopsidis,* and *Pseudomonas syringae*. Even the overexpression of *DkLOX3* exhibited high resistance to *Botrytis cinerea* and *Pseudomonas syringae* by correcting ROS accumulation and cell death in the *Arabidopsis thaliana* [107]. It was observed that the *LOX1* gene guard cells play a more considerable role in the stomatal defense response than ABA [108].

In tomatoes, mutations in *LOX1* were reported to weaken the process of stomatal closure, making it more prone to *Pseudomonas syringae* [8]. Moreover, higher activity of 9-LOX has been observed in *Arabidopsis thaliana* roots, where the products of 9-LOX adjust the construction of roots [40]. In potatoes, 9-LOX oxylipins participate in defense mechanisms against the initial stage of *Phytophthora infestans* [8]. The *Spodoptera exigua* larvae feeding on *Zea mays* induce the *9-LOX* expression to a higher level than 13-LOX or LOX3 upon the injection of *Aspergillus flavus* and *Fusarium verticillioides* [109]. The enhancement of LOX3 activity in rice leaves after *Magnaporthe grisea* infection was also reported. The *Osr9-LOX1* interacts with 13-LOX, assisting a pathway to provide resistance against chewing and piercing-sucking types of herbivores. The interaction of almond-*Aspergillus carbonarius*, 9-LOX generates 9-hydro (peroxy) fatty acids (HFAs) that are further metabolized into the C9 aldehydes HPL-branch pathway. In pearl millet, the intensity of LOX activity was augmented and connected with the extent of host resistance during interaction with the *Sclerospora graminicola* and in downy mildew-resistant crops. Similarly, infection of *Aspergillus flavus* and *Aspergillus parasiticus* in the peanut induces LOX, and consequently, various anti-microbial materials are synthesized through LOX feedback [1]. In *Zea mays*, *ZmLOX6* and *ZmLOX10* are two plastidial *13-LOX* genes stimulated by the action of fungus *Cochliobolus* *carbonum* under compatible interactions [15,110]. Responses of LOX3 and LOX4 to the *Heterodera schachtii* and *Meloidogyne javanica* in *Arabidopsis thaliana* seedlings were observed. They act differently, with distinct signaling and metabolic pathways. LOX4 performs a crucial role in directing plant defense against nematode infection [111]. Even the injection of pea seed-borne mosaic virus (PSbMV) in pea (*Pisum sativum*) shows enhanced activity of LOX [112]. Induction of *CsLOX1* is quick during the feeding of *Empoasca vitis* (tea green leafhopper), while feeding by *Toxoptera aurantii* (tea aphid) resulted in a pattern of induction and repression in the tea plant [113]. Seedlings of papaya demonstrated anti-fungal activity, by 13-LOX-generated hydroperoxides against the *Phytophthora palmivora*, via hampering germination of sporangia and mycelium growth [114]. Various isoforms of LOX were observed in potatoes during wounding, late blight, and other pathogen infections [8].

LOXs were reported only in Gram-negative bacteria *Pseudomonas aeruginosa* and *Myxococcus xanthus*, Gram-positive bacterium *Thermoactinomyces vulgaris*, and cyanobacteria *Anabaena*/*Nostoc* sp. PCC 7120, *Cyanothece* sp. PCC8801, *Nostoc punctiforme* PCC 73102, and *Acaryochloris marina*. Studies present the biological role of LOX in biofilm growth by modulating lipid signaling between the host and the pathogen during colonization of the host’s airway epithelium. LOX can act as a virulence factor during infections by the oxidation of membrane lipids (hemolysis). LOXs are also well-characterized enzymes, efficiently expressed by the microbial hosts, that increase the development of innovative biocatalytic processes for green leaf volatiles production [115]. It was observed that milk fermented by a *Lactococcus lactis* strain producing 15-LOX-1 has beneficial and avirulent roles, i.e., it reduced inflammatory activities and enhanced the immune system [116].

## 9. LOX Activity as a Molecular Marker for Stress Tolerance/Stress and LOX Activity

The activity of LOX is considered a good biological marker in the field of plant physiology [95,100]. Plants facing thermal stress, pathogen infection, and mechanical damage increase the activity of 13-LOX [47,48,49]. LOX activity can be further decreased by low temperature, ABA, retinoids, epoxide derivatives of linoleate, and polyamines [45]. Exposure of plants to salt stress activates LOX and oxidation of lipid peroxide. Hence, an increase in LOX activity, to some extent, was observed in the salt-tolerant plant facing salt stress [117]. Expression of the *LOX* gene was observed under low-temperature conditions, which indirectly indicates the involvement of LOX in the adaptive response of plants towards temperature stress [118]. Increased LOX activity under low temperature is linked with phospholipase D action, causing phospholipid degradation to release PUFAs—the LOX substrates [119]. The phosphatidic acid, a product of phospholipase D, directly modulated LOX activity when observed under in vitro conditions [120]. Cold and salt stress influence LOX activity in a different manner. At the early stage, salt stress reduced the LOX activity in *Zea mays* and had a near nil effect on 13-LOX activity; ultimately, the 9-LOX activity returns to the normal state, while the activity of 13-LOX gets reduced. The cold stress repressed 9-LOX activity, whereas 13-LOX activity was significantly enhanced, indicating two distinct LOX cascades in producing active products due to abiotic tension [121].

The change in LOX activity, facing a short-term hyper and hypothermia, was connected with the ecological policies of the individual plant taxon. Under control conditions, seedlings of stress-sensitive *Festucan pratensis* had the lowest pace of LOX activity and were distinguished by a remarkable drop in enzymatic activity after low and high-temperature exposure. In *Amaranthus caudatus* (a heat-resistant ruder plant), LOX activity was increased by 76% due to high temperature. LOX activity in *Brassics napus* seedlings disclosed that low temperature was responsible for reduced LOX activity by 34% in heat resistant cultivar, while subsequent heat stress gave no change to LOX activity. The LOX activity in heat-stressed cold-resistant cultivar was reduced two-fold; however, the cold stress resulted in no remarkable change [122].

Small-phase temperature stress was reported to influence LOX-activity in cultivars of *Triticum aestivum*. In contrast, their thermo-stability indicated that both high (+40 °C, 2 h) and low temperature (+4 °C, 2 h) uphold the LOX activity, witnessing the participation of the products of LOX cascade in providing defense and stability under thermal stress. Meanwhile, short-term exposure to high-temperature stress results in enhanced activity of all the 9-LOX isoforms in the cold-resistant cultivar. Still, the most significant increase is observed in LOX-1 (pH = 7.0), from the above-ground seedling part, and in 9-LOX (pH = 6.5) from the roots. LOXs and LOX-oxidation products of PUFAs play a crucial role in plant metabolism, affect plant growth and development, and affect plant capacity to tolerate various biotic and abiotic stresses. LOXs potentially participate in signaling caused by stressor effects, and the activity of LOXs efficiently serve as molecular markers for monitoring stress tolerance in plants [123].

## 10. LOX Activity and Their Products

The LOX family is responsible for the stereo and region-specific addition of O_2_ to the 1,4-*cis*,*cis*-pentadiene segment of linolenic acid, α-linolenic acid, and linoleic acid. A hydro-peroxide product of LOX activity, having a conjugated *cis*/*trans* complex, is produced via the relocation of a double bond during the catalytic cycle. Oxidation of linoleic acid is the initial step of a branching-enzyme cascade, leading to the production of oxylipins. They participated in developing plant responses against the consequences of various biotic and abiotic stresses and in regulating apoptosis and aging [28].

The content of PUFAs (the LOX substrates) is increased in stressed plants [46,52,98]. Generally, the LOXs found to oxidize linolenic acids at the C-9 or C-13 positions result in 9 or 13-HPODEs, which start around six enzymatic pathways. In plants, the biosynthetic pathway of LOX involves some parallel branches named according to the six enzymes of subsequent branches, such as AOC, AOS, divinyl ether synthase, HPL, epoxy alcohol synthase, peroxygenase, and others in which several biologically-active metabolites are synthesized. In the HPL pathway, 9-hydroperoxylinoleat is changed to C9-aldoacids and C9-aldehyde, as well as 13-hydroperoxylinolenat or 13-hydroperoxylinoleate to C12-aldoacids and C6-aldehydes. The ketolic pathway having AOS is used up by 13-LOX, and the AOC pathway leads to JA biosynthesis. The branch of divinyl ether is also recruited for producing 9 and 13-LOX, specifically etherolenic and colnellenic acids. Another branch of the LOX pathway is higher for synthesizing epoxy and hydroxyl-derivatives of PUFA through peroxygenase, i.e., synthesizing a monomeric substrates of heteropolymer cutin key component of the cuticle [51,95]. Both free-oxygenic fatty acids and fatty acids, as reserve galactolipids, phospholipids, and triacylglycerols, can be the LOX substrates. During the deoxygenation of PUFAs, the oxygen functions as a second substrate. Delivery of PUFAs and oxygen to the active center of LOX is supposed to be done by two hollows, but how and where they have been added to the active center was debatable. Though it was already displayed that electrostatic interaction between a carboxylic group of PUFAs and amino acid residues, with a positive charge of LOX, is a shaping factor in building the enzyme-substrate complex [124,125].

PUFA-hydroperoxide (product of LOX activity) is the hepoxilin and lipoxin predecessor. These synthesized oxylipins articulate enzymes and proteins synthesis, and the plant antibiotics participated in the detoxification of pathogen-attacked plants. JA is an important member of the oxylipin family [126]. JA is associated with the regulation of seed germination, development of pollen, methylene synthesis, tuber-genesis, plant aging [126], and with transmitting signals against biotic and abiotic stress effects [127].

Pathogenic infections and mechanical damages are responsible for marked improvement in some oxylipins’ contents, making it more convenient to recognize them in crop tissues. Infection of *Verticillium longisporum* to *Arabidopsis thaliana* resulted in high content of few compounds, including donor-OPDA, 9,12,13-trihydroxy-10-octadecanoic acid, and 9,12,13-trihydroxy-10,15-octadecadienoic acid, that boost the plant immunity [128]. Physiologically active oxylipins (2*E*)-dodecene-1,12-dicarboxylic acid (or traumatic acid), and 12-oxo-*trans*-10-dodecenoic acid (or traumatin) are also capable of inducing cell multiplication and callus formation at the damaged sites [45]. The disintegration of plant tissues escorted the discharge of volatile C6 and C9 compounds, recognized by a characteristic smell of freshly cut grass. Hexenals and hexenols (C6 compounds) are significant anti-fungal and anti-microbial agents that provide primary defense against attackers [35]. Tobacco (*Nicotiana tabacum*) infected with *Golovinomyces cichoracearum* resulted in an enhanced level of 2(*E*)-hexenal [129]. Infection of *Botrytis cinerea* boosted the (2*Z*) and (3*Z*)-hexenals, (3*Z*)-hexenilacetat, and (3*Z*)-hexenol level, which repressed the growth of fungal infection in tomato tissues [130]. The (2*E*) and (3*Z*)-nonenals (the volatile C9 compounds), with the fragrance of fresh cucumber, were secreted as a consequence of mechanical damage [131] and reduced the action of pathogens [132].

*LOX* genes expression is regulated by pathogens and phytohormones, i.e., the *AtLOX1* gene was induced by JA and ABA, and the *OsLOX3* gene was induced by JA [133]. Mutation in *AtLOX3* and *AtLOX4* led to developmental dysfunctions. The expression of *AtLOX2*, *AtLOX6,* and *TomLoxD* is related to wound-induced JA biosynthesis [134,135]. *OsLOX2* overexpression resulted in a reduced seed germination period [86]. *AdLOXs* of *Actinidia deliciosa* (kiwi) participate in developing fruit aroma [136]. On the other hand, the silencing of *CaLOX2* led to a reduced JA level and thrips resistance [137].

## 11. Conclusions and Future Perspective

LOXs are oxidoreductase enzymes found in plants and animals. These are the assemblage of non-heme iron and manganese-containing dioxygenase redox enzymes, having two distinct domains, with a molecular weight of 90–110 kDa. LOX catalyzes PUFAs and esters with hydroperoxide derivatives; *cis* and *trans*-pentadiene structures, with conjugated double bonds, are synthesized by adding intra-molecular oxygen. In plants, linoleic and linolenic acid act as LOX substrates. LOX mediates JA biosynthesis and acts as the stress biomarker against fungal, bacterial, pest, and abiotic stresses (salt, heat, cold, drought, and radiation). Furthermore, LOX also induces seed germination, growth, and male gametophyte formation. Unraveling the contribution of LOX in plant physiological processes is essential to be explored. Further investigations must be carried out on the mechanism of LOX with phytohormones (e.g., auxins, BRs, JA, and cytokinins), during plant growth and development, in stress mitigation. Studies on molecular mechanisms in stress amelioration shed new light on the alteration in plant physiology and biochemistry.

## Figures and Tables

**Table 1:** Genes encoding LOX in *Arabidopsis thaliana* [38,39,40,41,42].

Gene Locus	Gene Name	Gene Function	Total Amino Acids
AT1G55020	lipoxygenase 1 (LOX1)	conferring resistance pathogens	859
AT3G45140	lipoxygenase 2 (LOX2)	biosynthesis of JA	896
AT1G17420	lipoxygenase 3 (LOX3)	flower development, catalyze the oxygenation of fatty acids	919
AT1G72520	lipoxygenase, putative (LOX4)	flower development, 13-lipoxygenase induced by abiotic stresses, triggers defense responses	926
AT3G22400	lipoxygenase 5 (LOX5)	defense response	886
AT1G67560	lipoxygenase family protein (LOX6)	biosynthesis of JA, PLAT/LH2 domain-containing lipoxygenase family protein	917

**Table 2:** Genes encoding LOX in *Oryza sativa* [44].

Gene Locus	Gene Function	Total Amino Acids	Chromosome Number
LOC_Os11g36719	lipoxygenase, putative, expressed	869	11
LOC_Os12g37260	lipoxygenase 2.1, chloroplast precursor, putative, expressed	923	12
LOC_Os12g37320	lipoxygenase 2.2, chloroplast precursor, putative, expressed	359	12
LOC_Os02g10120	lipoxygenase, putative, expressed	927	2
LOC_Os02g19790	lipoxygenase 4, putative, expressed	297	2
LOC_Os03g08220	lipoxygenase protein, putative, expressed	919	3
LOC_Os03g49260	lipoxygenase, putative, expressed	868	3
LOC_Os03g49380	lipoxygenase, putative, expressed	878	3
LOC_Os03g52860	lipoxygenase, putative, expressed	871	3
LOC_Os04g37430	lipoxygenase protein, putative, expressed	798	4
LOC_Os05g23880	lipoxygenase, putative, expressed	848	5
LOC_Os06g04420	lipoxygenase 4, putative	126	6
LOC_Os08g39840	lipoxygenase, chloroplast precursor, putative, expressed	925	8
LOC_Os08g39850	lipoxygenase, chloroplast precursor, putative, expressed	942	8

## Data Availability

Not applicable.

## Abbreviations

10-OPEA	10-oxo-11-phytoenoic acid
AA	amino acid
ABA	abscisic acid
AOC	allene oxide cyclase
AOS	allene oxide synthase
BR	brassinosteroid
DES	divinyl ether synthase
ET	ethylene
HFA	9-hydro (peroxy) fatty acid
HPL	hydroperoxide lyase
HPO	hydroperoxy fatty acid
9-HPODE	(9*S*,10*E*,12*Z*)-9-hydroperoxy-10,12-octadecadienoic acid
13-HPODE	(13*S*,9*Z*,10*E*)-13-hydroperoxy-9,11-octadecadienoic acid
ICK1	inhibitor/interactor of cyclin-dependent kinase 1
JA	jasmonic acid
LA	α-linoleic acid
LOX	lipoxygenase
MeJA	methyl jasmonate
OPDA	*cis*-12-oxophytodienoic acid
PAL	phenylalanine ammonia-lyase
PSbMV	pea seed-borne mosaic virus
PUFA	polyunsaturated fatty acid
VSP	vegetative storage protein
